# Ready, Set, Clerkship: The ‘Learning to Learn at the Workplace’ Training to Prepare Medical Students for Workplace Learning

**DOI:** 10.5334/pme.1567

**Published:** 2025-06-10

**Authors:** Mila Van Dorst, Marije Lesterhuis, Renske De Kleijn, Annet Van Royen-Kerkhof, Marije Hennus

**Affiliations:** 1 Department of Education of the Wilhelmina Children’s Hospital, University Medical Center Utrecht, Utrecht, NL; 2 Utrecht Center for Research and Development of Education, University Medical Center Utrecht, Utrecht, NL; 3 Wilhelmina Children’s Hospital, NL; 4 Utrecht University/University Medical Center Utrecht, Utrecht, NL; 5 University Medical Center Utrecht, Utrecht, NL

## Abstract

**Background:**

Preparing medical students *how* to learn during clerkships is vital to support their transition from preclinical to workplace learning. However, training programs fostering students’ workplace learning skills are sparse. To address this gap, the ‘Learning to Learn at the Workplace’ training program was developed, combining strategies for informal workplace learning with self-regulated learning (SRL) techniques.

**Approach:**

Using the ‘ADDIE’ instructional design model, the training was developed, implemented and evaluated. It consists of four classroom sessions combined with workplace assignments, each addressing a different SRL theme for informal workplace learning: learning goals, asking questions, feedback, and reflection. Additionally, the influence of the clerkship context on SRL is addressed, with the aim to enable students to recognize and utilize informal workplace learning.

**Outcomes:**

The training was piloted with medical students during their second or third clerkship (n = 33). Students provided written feedback following each session and completed a final questionnaire (n = 21). Teachers evaluated implementation fidelity after each session. Students reported that the training effectively supported their self-regulated workplace learning. They particularly valued its relevance, practical tools, and the opportunity to exchange clerkship learning experiences.

**Reflection:**

Students gained valuable insights into self-regulated informal workplace learning at clerkships. To further stimulate application of taught skills during clerkships, better integration of transfer tasks into the training is suggested. Involving numerous stakeholders and extensive literature in the developmental process ensured this training aligned with students’ needs and received positive evaluations from students, teachers, and curriculum developers. Subsequently, the training will become a mandatory part of the current curriculum.

## Background & Need for Innovation

Clerkships are a significant component of the medical curriculum, where students are expected to learn from engaging in patient care, often referred to as workplace learning (or clinical/experience-based learning) [1]. Workplace learning is distinctly different from preclinical learning in classrooms which is more organized. Not surprisingly, the transition from preclinical to workplace learning is a well-known challenge for medical students [1,2,3]. Students are often overwhelmed during their first clerkships [3]. They need to deal with the unpredictability of their day: for example, who is my supervisor today; what will I encounter at the ward, clinic, or emergency department; what is expected from me in this specialty? Moreover, in clinical practice, patient care has priority over learning, which results in supervisors having little time to support learning. This places a greater responsibility on students to regulate their own learning [1,2,3,4], and thus the call to prepare students for workplace learning [2,5,6].

Most workplace learning occurs informally, meaning learning from the work itself or from others at, or during work [1,7,8]. It involves learning by doing, observing, or discussing. Eraut defines informal learning as “learning that comes closer to the informal end than the formal end of a continuum” (Eraut, 2004, p250). On this continuum, ranging from highly to less informal, Eraut distinguishes three levels of intentions to learn informally; *implicit*, being unaware of learning, *reactive* a more consciously on the spot learning, and *deliberative* which entails planning of and engaging in activities to learn from [8]. We propose that the first step in preparing students for workplace learning is to enhance their ability to recognize learning opportunities by raising their awareness of informal learning during clinical activities. Next, we suggest improving their skills to effectively utilize ad-hoc opportunities (reactive learning) and to purposefully plan, engage in, and reflect on learning opportunities (deliberative learning). The theory of self-regulated learning provides valuable insights on how students could achieve this.

Self-regulated learning (SRL) involves goal setting and planning; engaging in strategies to achieve and monitor these goals, and reflecting [9]. Medical students are accustomed to self-regulating their learning during preclinical learning, with guidance from the medical curriculum. However, when transitioning to workplace learning during clerkships, they must adjust these SRL skills to be effective [4,5,6]. To aid in this adjustment, training that specifically supports SRL development for workplace learning is crucial [2,4,5,6]. Despite this need, few studies have explored interventions aimed at enhancing students’ learning skills to facilitate their adaptation to workplace learning in clerkships [2,10,11,12]. To fill this gap, we propose a training program that focuses on students’ learning skills only and distinguishes itself from other interventions by its explicit focus on informal learning and the skills informal learning requires. By incorporating informal workplace learning with SRL strategies, we aim to strengthen students’ ability to learn effectively and seek learning support from supervisors and colleagues during their clerkships.

## Goal of Innovation

As a primary goal, the ‘Learning to Learn at the Workplace’ training program was developed to support medical students’ transition to workplace learning. This training provided knowledge and tools to enhance students’ skills in recognizing, utilizing, and reflecting on learning opportunities during clerkships and seeking support from their workplace supervisors. A secondary goal was to stimulate students’ application of training content during clerkships as effective training must result in functional application in authentic contexts [13]. The final goal was sustainable integration of this training into the medical curriculum, ensuring its long-term impact.

## Steps taken for Development and Implementation of Innovation

The training was developed, implemented, and evaluated, using the ADDIE instructional design model including the phases: Analysis, Design, Development, Implementation, and Evaluation [14]. In all phases, stakeholders and relevant literature were incorporated.

### Analysis

Semi-structured interviews (n = 25) with stakeholders affiliated with the University Medical Center Utrecht (UMC Utrecht) were conducted to inventory students’ problems and needs with respect to learning during clerkships (see Supplement for the interview guide). Stakeholders included: six medical master’s students (from the first, second, and final year); six recently graduated doctors; four clinical supervisors; four clerkship coordinators; two coordinators of students’ professional and personal development courses, and one bachelor and two master coordinators. Stakeholders’ concerns aligned with extant literature and yielded five main themes for the training (see Table 1). The themes are the overarching theme of informal workplace learning, and four SRL themes: learning goals, asking questions, feedback, and reflecting. All stakeholders expressed their desire for training for students on effective learning during clerkships.

**Table 1:** Outcomes of literature and stakeholder analysis.


CONCLUSIONS FROM LITERATURE ANALYSIS	DESCRIPTIONS OF EXAMPLES MENTIONED IN STAKEHOLDER INTERVIEWS	RESULTING TRAINING THEMES

Students starting their clerkships have insufficient insights into informal workplace learning and how to effectively apply SRL skills in a clerkship context [1,2,3,4]	E.g., students not recognizing observing as a learning opportunity	Informal workplace learning;
	E.g., students not knowing when it is approriate to ask questions or what to ask	Asking questions;

Students’ awareness of how the clerkship context influences their SRL is limited [5,6]	E.g., students being unable to set realistic goals, not yet knowing the main activities of their clinical workplace or with whom to discuss their learning goals	Informal workplace learning; Learning goals;

Students need to take more agency in SRL and adapt to little supervisor time [1,2,3,4,5,6]	E.g., students being reluctant to ask for feedback on their develomental areas (prioritizing performing over learning) and having to adjust to (also) take the initiative in feedback processes by asking for feedback from their busy supervisors	Informal workplace learning; Feedback;
	E.g., students (and supervisors) experiencing to have/make insufficient time to reflect on what is being learned during their busy day	Reflecting;


### Design

To ensure the program met students’ needs, the training content was designed based on theories related to the five identified themes, Eraut’s theory of informal workplace learning and self-regulated learning theory [8,9,15]. Blume’s theory on promoting training transfer was also applied [13]. In his Dynamic Transfer Model, Blume outlines different phases within the transfer process (i.e., intention, initial attempt and evaluation of initial attempt) and how contextual and individual characteristics influence these processes and their outcomes. When moving through these transfer processes, self-regulatory mechanisms are applied [13]. Furthermore, stakeholder input was sought during three design sessions. Each two-hour design session included approximately 10 strategically selected stakeholders (a combination of students, teachers and coordinators of medical/nurse students’ professional and personal development courses, clinical supervisors, clerkship coordinators, and the master coordinator). These sessions were facilitated by the training program development team, consisting of three recently graduated doctors, a medical specialist and two educational scientists. During the design sessions, stakeholders and the training development team collaboratively translated the five main training themes from the analysis phase into learning objectives and developed realistic cases for case-based exercises and workplace assignments. All educational materials (in Dutch) are openly accessible via this open education resources platform (see Supplement for learning objectives and examples of educational material in English).

### Development

**Figure 1:**
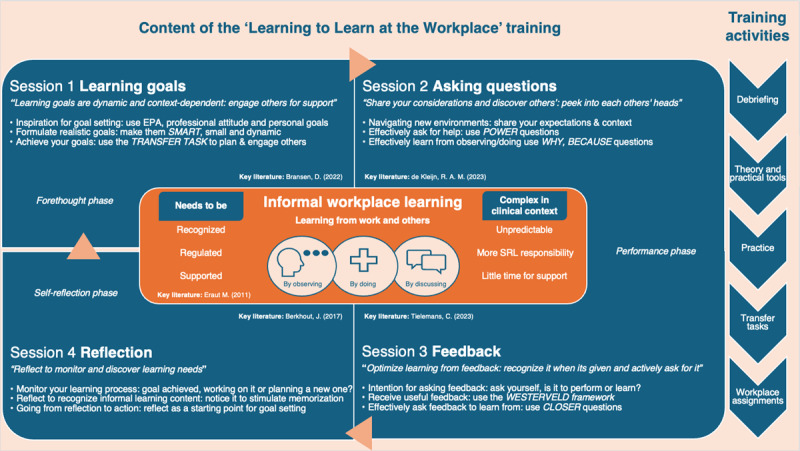
**Overview of the ‘Learning to Learn at the Workplace’ training**. The main training content is highlighted for each session, including key literature [5,6,15,16,17] that shaped the theory and practical tools. Practical tools used are indicated in capitals and italics. The cyclical process of self-regulated learning is visualized through arrows, applying Zimmerman’s phases of forethought, performance and self-reflection [9]. List of abbreviations; SRL: Self-Regulated Learning; EPA: Entrustable Professional Activities; SMART: Specific, Measurable, Achievable, Relevant, and Time-based; POWER: Problem, Options, Weighing options, Expressing preferred option, Request; CLOSER; Current performance, Learning Objective, Self-Evaluation, Request.

Training content was refined based on the outcomes of the design phase and key literature on the five themes (Figure 1) by the training program development team. The resulting training included four three-hour sessions centered on recognizing and participating in informal workplace learning and engaging others to support this process (Figure 1). Each session focused on a main SRL theme to regulate informal workplace learning. Barriers and facilitators related to the SRL theme in the clerkship context were addressed by encouraging students to share their clerkship learning experiences and strategies with other students.

Each training session consisted of various activities during the session, the transfer task, workplace assignments, and a debriefing in the subsequent training session (see training activities, Figure 1). During interactive discussions, students and teachers discussed their experiences with the SRL skill addressed in that session – for example asking questions during clerkship – which teachers then connected to relevant theory (e.g., sharing what you already know in a question, a POWER question, can help supervisors provide more targeted help [16]). Students subsequently practiced POWER in a case-based simulation and discussed their experiences. Aligning personal experiences with theory and providing opportunities to practice with a tool during the training was intended to lower barriers and motivate students to implement the SRL skills and tools during the clerkship. Transfer tasks included prompts to support students’ reflection at the end of a session on what training content they intend to apply during the clerkship, why/how they intend to apply this SRL skill, who in the clerkship could support them, and what potential barriers and coping strategies they foresee when applying this SRL skill in their current clerkship. In addition to setting their own concrete goals, students received workplace assignments to inspire and further encourage the application of SRL skills during the clerkship (e.g., practice with asking workplace supervisors POWER questions at three different times). In addition to stimulating students’ implementation of SRL skills, transfer tasks and workplace assignments were also intended to stimulate students to actively engage their workplace supervisors in this process (coregulation) [13]. Finally, to stimulate students to evaluate their application of SRL skills in the clerkship, each subsequent session began with a small group and plenary debriefing where students reflected on their progress towards their transfer task goals and shared clerkship experiences related to the SRL theme discussed.

### Implementation

In 2023 the training was piloted on a voluntary basis among UMC Utrecht medical students in their second or third clerkship (i.e. first year of the medical master’s program). This timing was chosen based on stakeholder input, indicating that students require prior clinical experience to be motivated for this training. Enrollment conditions included:

– Being enrolled in clerkships during the training
– Full-time student status
– Anticipated 100% training attendance

Recruitment was conducted through pitches, flyers, and the faculty newsletter resulting in 42 of 70 targeted students (60%) applying. Eventually, 33 students started the program. Withdrawals were due mainly to strict enrollment conditions. Students were divided into two groups, each led by a recently graduated doctor and either an educational scientist or a medical specialist/educator with extensive expertise in informal workplace learning. The development team deliberately paired recently graduated doctors, who had recent clerkship experiences, with experienced educators to enhance students’ perceptions of the credibility and practicality of the training, thereby stimulating training transfer [13].

Of the 33 students, 16 attended all sessions (48%) and 24 at least three sessions (73%). Most dropouts were after the first session (n = 7, 21%), due to prioritizing clinical learning opportunities and clerkship continuity, followed by illness or practical issues.

## Evaluation of Innovation

Training effectiveness was evaluated by assessing implementation fidelity [18], identifying training successes (i.e., achievement of intended goals), and pinpointing areas for improvement. Teachers used implementation fidelity forms directly after each session to evaluate whether the training was delivered as designed and how they perceived students received it (see Supplement for a fidelity form). Furthermore, students provided initial feedback via anonymous post-its at the end of each session and completed an evaluation form at the program’s conclusion (including 5 and 3-point scale items and text boxes for narrative feedback, see Supplement for the evaluation form). The initial feedback was discussed by teachers and developers after each session to refine subsequent sessions.

### Teacher feedback to assess implementation fidelity

Deviations from the intended program occurred primarily in the first two sessions, including more interactive activities and shortening the training by 30 minutes. These changes were made because the training followed a half day of clerkship, leading to waning concentration. The alterations improved students’ engagement during the sessions and prevented further dropout. Additionally, the medical specialist teacher shared supervisory perspectives with both groups, which enhanced the credibility of training content as perceived by students. Moreover, the teachers suggested introducing the training earlier in the curriculum as they often had to ‘unteach’ unhelpful coping behaviors students had developed during previous clerkships.

### Student feedback to identify training successes and areas for improvement

**Figure 2:**
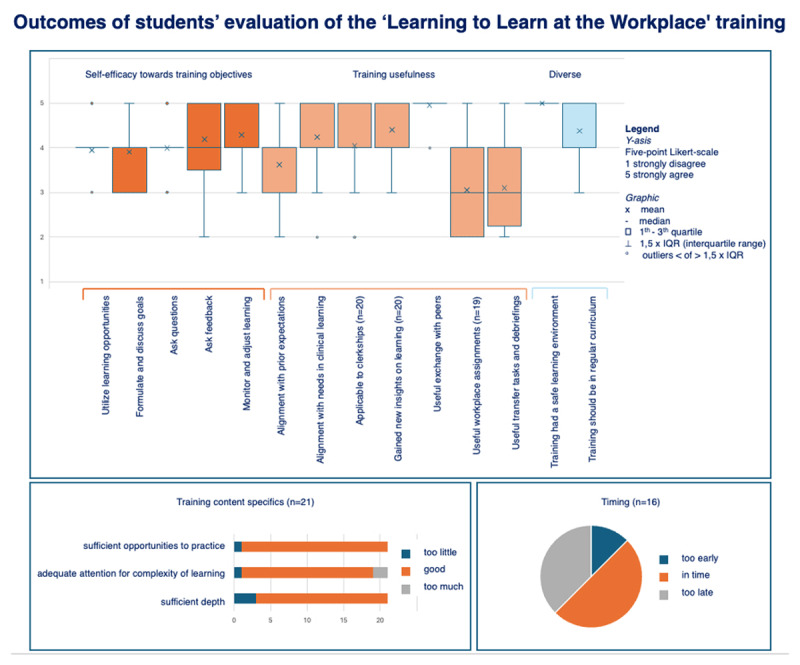
**Evaluation outcomes of the ‘Learning to Learn at the Workplace’ pilot**. Visualization of student evaluation form data, including students’ self-efficacy of the main trainings’ learning objectives, its usefulness, content specifics and timing in the curriculum. The number of responses per item was 21 unless stated otherwise.

The evaluation form was filled in anonymously by 21 students. Students reported increased confidence in all five training objectives (Figure 2). They agreed that training content was well aligned with their learning needs during clerkships and the practical knowledge and tools were highly applicable to their clerkship settings (e.g., pocket cards [16,17]). Students valued the safe learning environment of the training program that encouraged sharing workplace learning experiences and strategies with peers and teachers (Figure 2). Many expressed feeling validated and less isolated in facing clerkship challenges:

*“…the training shifted my focus to being here [at clerkship] to learn, and as a result I learned more and experienced less stress”*.

Students also appreciated having a medical specialist and young doctors as teachers, who understood the clerkship experience and provided a supervisor’s perspective. Interactive and diverse activities (e.g., podcasts, simulations, and games) were particularly well-received after adjustments were made following the initial session. Finally, students were in favor of introducing the training earlier in their curriculum, as was suggested in the narrative feedback:

*“…earlier, but not before the start of a clerkship”*.

The transfer tasks were perceived to be less useful or unclear, as were the workplace assignments that were often forgotten by students because they were insufficiently covered in the training itself (Figure 2). Additionally, case-based exercises were sometimes perceived as unrealistic or too lengthy, with students preferring personal or more varied examples. Repetition of content or exercises (e.g., formulating SMART goals) across sessions was suggested in the narrative feedback. Finally, some students indicated that it remained challenging in specific situations to ask for support during clerkships (e.g., difficult to discuss learning goals or feedback when they perceived their workplace supervisors had no time).

## Critical Reflection on the Process

Our primary goal, creating awareness among students about informal workplace learning and providing them with learning skills to regulate their workplace learning during clerkships, was met. In an effort to achieve our second goal, multiple transfer principles were followed to enhance students’ perceived credibility, practicality and need of training content and thus their intention to apply the SRL skills at clerkship [13]. For instance, the training used applicable tools, teachers and students shared examples from their clinical workplace experience, and many opportunities were provided for students to self-reflect on their learning during clerkships. Moreover, transfer tasks, workplace assignments, and debriefings scaffolded students through the transfer process: formulating concrete intentions, stimulating initial attempts to apply SRL skills at clerkship, and evaluating these attempts. Guiding students through this process is crucial, as the (evaluation of their) initial attempt influences the decision to continue applying these skills [13]. Although students reported having learned a lot about informal workplace learning in a clerkship context, the transfer tasks and workplace assignments were perceived as less valuable. Consequently, students may not have discussed and practiced setting learning goals, receiving feedback, asking questions and reflection in coregulation with their supervisors at the workplace. This raises concerns about the extent the training changed students’ behavior in the short and long term. Because the training was designed to evaluate students’ actual application of SRL skills during (small) group debriefings, we only collected anecdotal examples of students’ (un)successful implementation of SRL skills at clerkship. Therefore, we propose future research on training transfer to gain deeper insights into students’ decisions to apply or not (continue to) apply SRL skills during clerkships, and how training design, personal or contextual clerkship factors influence these decisions [13]. These insights would benefit this and other training programs by enhancing interventions such as transfer tasks or workplace assignments to better stimulate students’ implementation of skills at clerkship. Additionally, we suggest that future iterations of the training should place greater emphasis on executing tasks that stimulate transfer. For example, transfer tasks or workplace assignments could be made more relevant by increasing their significance in follow-up training days or by linking them to common clinical routines or interactions with workplace supervisors. Nevertheless, the content of this training was positively valued by students and has triggered a change in how they viewed learning during clerkships. More specifically, they came to understand that learning challenges are inherent to learning during the clerkship because of the complexity of workplace learning in that context and that they are not alone in these challenges. This contributed to students’ self-efficacy and feelings of empowerment in their learning during clerkships. However, to what extent they will apply this in subsequent clerkships remains to be further investigated.

Key to the developmental process of this training was the balance between strong stakeholder involvement and theoretical input. This resulted in a highly relevant training for students, which is now being embedded in the mandatory curriculum, thereby achieving our third goal for this initiative. On the other hand, it is important that we acknowledge that participation during the development and implementation of the training was voluntary. The stakeholders who contributed during the developmental phase and students who voluntarily engaged in the training may have already perceived the topic as important. Consequently, this could have influenced the evaluation results. As the training is now being integrated into the current mandatory curriculum, it is essential to evaluate how it is received by potentially less motivated students. Additionally, success of the training also depends on the alignment with the workplace. For example, when students attempt to discuss their learning goals but their supervisor does not allocate time, students may quickly abandon this behavior. Therefore, success of this training also depends on broader initiatives to improve the workplace learning environment. For example a broader initiative might be training the workplace supervisors to enhance their skills to guide students’ workplace learning (e.g., the interprofessional Clinical Teaching Qualification (CTQ) program [19]). A shared mental model among students and their workplace supervisors regarding informal workplace learning and SRL could further optimize training success. Such a shared understanding can create a supportive learning environment that facilitates engagement in workplace learning [8,15] and the actual transfer of skills to the workplace [13].

The ‘Learning to Learn at the Workplace’ training addresses a recognized need among stakeholders, not in the least the students themselves, for support in transitioning to workplace learning during clerkships. By building on theories of informal workplace learning and self-regulated learning, the training enhances students’ readiness to learn effectively in a complex clinical environment. Evaluation results have informed its incorporation into the current curriculum, with plans to expand its role in the upcoming revised curriculum.

## Supplementary data

10.5334/pme.1567.s1 Including the stakeholder interview guide; the trainings’ learning objectives and examples of educational material; an example of a fidelity form; the students’ evaluation form.
