# Supplementary material for: Ready, Set, Clerkship: The ‘Learning to Learn at the Workplace’ Training to Prepare Medical Students for Workplace Learning
Source: Perspect Med Educ. 2025 Jun 10;14(1):352–9. doi: 10.5334/pme.1567 (PMC12164758; doi:10.5334/pme.1567)
Supplement: Supplementary file 1 — Including the stakeholder interview guide; the trainings’ learning objectives and examples of educational material; an example of a fidelity form; the students’ evaluation form. [file pme-14-1-1567-s1.pdf]

Supplementary data

Including:

- Stakeholder interview guide (p. 2-4)
- The trainings' learning objectives (p. 5-6) and examples of educational material (p. 7-13)
- Example of the fidelity form (p. 14)
- The students' evaluation form (p. 15-16)

Stakeholder interview guide
Stakeholder interviews were conducted in Dutch and translated into English to serve as Supplementary data for this article. A distinction in questions was made between three stakeholder groups: students and recently graduated doctors; clinical clerkship supervisors; clerkship coordinators, course coordinators, and bachelor and master program coordinators.

1. How do medical students learn during their clerkships?

Students and recently graduated doctors

- Describe what a typical day during your clerkship looks like. What are your normal daily activities? Think back to the previous week, were there any differences in activities that week, and if so, how was the work different?
- How did you learn to do what you now do in daily practice during your clerkship? *Help: Was this mainly informal, such as by observing and doing? Or formal, through organized and planned education? Or both?*
- From which situations/moments during your clerkship did you learn the most?
- From whom did you learn to do what you now do in daily practice during your clerkship? *Help: Clerkship supervisor, daily supervisor, nursing staff?*
- To whom do you ask for help or advice, and from whom do you receive it during your daily activities? Do you get enough help/advice? Are you able to ask for enough help/advice? *Help: Time, availability, barriers to contact*
- How do you take an active role in learning during your clerkship as a student?

Clinical clerkship supervisors

- What does the content of a clerkship look like for a medical student? What are the intended normal daily activities of the medical student?
- What knowledge, skills, and attitudes does a medical student need to optimally learn from daily work during a clerkship?
- What organized learning moments are there for medical students (formal learning)?
- How do you guide medical students in learning from their daily tasks during the clerkship (informal learning)? *Help: Opportunities for asking questions, just-in-time feedback, encouraging reflection, asking about learning goals, offering challenging but not overly complex tasks.*
- Who is involved in the daily guidance and support of medical students to enhance their learning during the clerkship?

Clerkship Coordinators, Course coordinators, and Bachelor/Master program coordinators

- What knowledge, skills, and attitudes does a medical student need to optimally learn from (the daily work during) a clerkship?
- What organized learning activities are clerkships required to offer?
- What agreements have been made about the daily guidance of medical students by clerkship supervisors to optimally support their learning during daily tasks in the clerkship?
- What information and/or education does the medical program provide about how to learn from daily activities during a clerkship (informal learning)?

2. What preparation precedes learning during clerkships?

Students and recently graduated doctors

- Describe how you were prepared for learning during a clerkship. What was the content of this preparation? Who provided this preparation and when?
- Have you received specific education or guidance on how to learn at your workplace?
- What did you feel unprepared for when you (just) started your clerkships? What preparation would you have liked to have?

Clinical clerkship supervisors

- Are students well-prepared for how they can learn during their clerkship?
- Do you actively prepare students for how they can learn during a clerkship? *If so, what is the content of this workplace learning preparation? Could it be improved? If not, how would you like to prepare students for workplace learning?*
- What guidance do you offer students to support their workplace learning during the clerkship? *Help: Encouraging the discussion of mistakes, reflection, feedback, actively involving and asking about learning goals and progress.*
- What preparation do you receive as clerkship supervisors for preparing and guiding students in learning during a clerkship? What preparation do you provide to other involved parties in guiding medical students in daily practice?

Clerkship Coordinators, Program Directors, and Teachers

- Are students well-prepared for how they can learn during a clerkship?
- How could the program better prepare students for learning during a clerkship?
- How could the program better prepare clerkship supervisors for guiding students in learning during a clerkship?

3. What factors influence effective learning during a clerkship?

Students and recently graduated doctors

- Describe how the context of your clerkship affects your learning. What factors stimulate your learning and what factors hinder effective learning during your clerkship? *Help: Time, support and guidance, task complexity, responsibility, team feeling.*
- What other factors stimulate or hinder effective learning during your clerkship? *Help: Individual factors, type of learning activities.*
- What aspects contribute to a good learning environment according to you?

Clinical clerkship supervisors

- How do you actively contribute to a good learning environment in your hospital as a clerkship supervisor?
- What factors stimulate or hinder effective guidance of students during their clerkship? *Help: Time, type of learning activities and (intensity of) guidance, who provides guidance.*
- How is time made for the guidance and support of medical students?

Clerkship Coordinators, Program Directors, and Teachers

- How do you promote a good learning environment for a clerkship from the program's perspective? And how is the learning environment of a clerkship monitored?
- How does the medical program help students and clerkship supervisors to reduce factors that negatively affect workplace learning? How does the medical program encourage students and clerkship supervisors to enhance factors that positively affect workplace learning?

4. Could a course on workplace learning help to learn more effectively during clerkships and later as a resident/specialist?

Students and recently graduated doctors

- What knowledge, skills, and attitudes are needed to learn from the daily work you perform during clerkships?
- How could you acquire the necessary knowledge, skills, and attitudes for workplace learning?

- As a medical student, but increasingly later as a resident/specialist, you are expected to contribute to the learning process of others. Besides learning how you as a student learn workplace learning, it is also relevant to learn how you can help others in learning at the workplace. Are you aware of this educational role?
- Support and guidance are very important for effective workplace learning. Who else do you think should receive education on how to guide others in workplace learning? Clerkship supervisors, daily supervisors (residents, nurse practitioners/physician assistants), nursing staff?

<u>Clinical clerkship supervisors, clerkship coordinators, course coordinators, and bachelor/master program coordinators</u>

- What knowledge, skills, and attitudes do students need to learn from the daily work they perform during clerkships?
- How could you teach the necessary knowledge, skills, and attitudes for workplace learning? What basic skills should (beginning) medical students have to learn efficiently at the workplace? How can you ensure they make the most of the clerkship and learn more from it? What is needed for this? *Preparation of students for workplace learning? Education of clerkship supervisors on guiding workplace learning? Reducing hindering contextual factors? Encouraging factors that enable workplace learning?*
- What knowledge, skills, and attitudes do you need as a clerkship supervisor to guide students in workplace learning?
- How could you acquire the necessary knowledge, skills, and attitudes for guiding workplace learning?
- Who should receive education on how to guide others in workplace learning? Clerkship supervisors, daily supervisors (residents, nurse practitioners/physician assistants), nursing staff, students themselves?

The ‘Learning to Learn at the Workplace’ learning objectives
Learning objectives were translated into English to serve as Supplementary data for this article.

Overarching learning objectives
At the end of this training:

- Is explained (using the sushi belt metaphor) why and how learning in the workplace during clerkships is complex and different from preclinical learning at university and why personal agency is important in workplace learning
- Has been explained that both students’ own behavior and the context at clerkships can influence learning
- Is explained why learning goals, feedback, asking questions, and reflecting are important for workplace learning within the clerkship context and why and how students can take responsibility for this themselves during clerkship

Learning objectives per theme

<u>Theme Informal Workplace Learning</u>
At the end of this training:

- Can the student inventory how, where, when, and from/with whom they can learn at their clerkship setting during their daily work (informal learning) using the Transfer Tasks, and adjust their learning to this during clerkship
- Can students effectively initiate, regulate and evaluate their learning during clerkships using learning goals, feedback, asking questions, and reflection
- Did the student practice in simulations during training sessions how to deal with some complex clerkship learning situations
- Can students monitor their own professional development and well-being by assessing what and how they want to learn this clerkship and by reflecting on how their clerkship affects them and their learning

<u>Theme Learning Goals</u>
At the end of this training:

- Can the student formulate (personal) learning goals that are context relevant and achievable using the Transfer Task, and apply and adjust these during clerkship
- Can the student effectively discuss (personal) learning goals with their supervisor or other relevant persons in their clerkship
- Can the student evaluate the way in which the formulation, discussion, application, and adjustment (if any) of the (personal) learning goals proceeded during the clerkship, paying attention to their own role in this and the role of their clerkship context

<u>Theme Asking Questions</u>
At the end of this training:

- Can the formulate a context-relevant question for an explanation of what they see/do during clerkship or ask for help with learning
- Can the student effectively seek clarification or assistance from the supervisor or other relevant individuals at their clerkship
- Can the student evaluate the way in which the explanation and/or request for help went during their clerkship, paying attention to their own role in this and the role of their clerkship context

Theme Feedback
At the end of this training:

- Can the student formulate a feedback question about a (personal) learning goal aimed at professional development and apply and adjust this during their clerkship
- Can the student effectively request, discuss, or give feedback to the supervisor or other relevant individuals at their clerkship
- Can the student evaluate feedback received and convert it into action (or not)
- Can the student evaluate the way in which the formulation, discussion, application, and adjustment or non-adjustment of the feedback question proceeded during their clerkship, paying attention to their own role in this and the role of their clerkship context

Theme Reflection
At the end of this training:

- Can the student formulate a reflection question about an observation, action, or experience during their clerkship to create a conscious learning opportunity and apply and adjust this during their clerkship
- Can the student formulate a reflection question aimed at evaluating their own learning and well-being in the complex clerkship context and apply and adjust this during their clerkship
- Can the student effectively reflect alone and with other relevant individuals at their clerkship context based on their own or others' observations, actions, and experiences during clerkships
- Can the student evaluate how they reflected during their clerkship, paying attention to their own role in this and the role of their clerkship context

Some examples of educational material

Educational material was translated from Dutch into English.

Session 1. Group Work: Learning activities at clerkship

Think back in your group to a normal day at clerkship. Discuss the clinical activities at your clerkship and what you can do to learn from these with your group and fill this table with your own examples.

From which clinical activities at your clerkship can you learn?	What can you do to learn from these activities?	For which activities you can learn from, does working stops?
**Participating in group discussions**<br>*MDO (multidisciplinary consultation meetings, i.e. "multidisciplinair overleg"), handover, ward rounds*	**Asking questions**	**Being supervised**<br>*Being observed or discussing patient details*
**Working alongside others**<br>*Doctors' room or nurse station*	**Looking up information**	**Being coached**<br>*Receiving explanation or discussing learning goals*
**Consulting**<br>*Consulting a doctor from a different specialty*	**Locating resource people**	**Being guided**<br>*Being assessed or discussing development*
**Participating in complex roles/tasks**<br>*Preparing a handover*	**Observing and listening**	**Shadowing**<br>*Shadowing someone during ward rounds/clinic*
**Problem solving**<br>*Differential diagnosing*	**Reflecting**	**Formal education at university**<br>*"terugkomdag"*
**Trying things out**<br>*Trying out a ward round structure*	**Learning from mistakes**	**Formal education at clerkships**<br>*Bedside teaching, scenario training*
**Skills training**<br>*Physical examination*	**Asking and discussing feedback**	**Assessment**<br>*Discussing an EPA (entrustable professional activities)-assessment*
**Working with patients**<br>*Interaction with (own) patient*	**Using mediating artefacts**	**Self study**

Preparing for plenary discussion during the training session

Eye openers. What clinical activities at clerkship didn't you consciously use yet but did you discover while talking (part 1 table) & what can you do during such an activity to learn (part 2 table) or should you put work on hold to learn this (part 3 table)?

From which clinical activities at your clerkship can you learn?	What can you do to learn from these activities?	For which activities you can learn from, does working stops?

Barriers: What clinical activities at clerkship would you like to use, but is difficult to do in practice (part 1 table) or what that you can do during such an activity to learn proves to be difficult (part 2 table) or does it proves difficult to stop your own work - or that of your supervisor (part 3)?

From which clinical activities at your clerkship can you learn?	What can you do to learn from these activities?	For which activities you can learn from, does working stops?

Session 2. Ask POWER questions (1)

The teachers discuss how asking POWER questions can assist students in receiving the help they need as the POWER question guides students to share their considerations. An example of a POWER question is being discussed with the students and subsequently they practice formulating POWER questions during a simulation exercise. *Example: I have received the blood test results of my patient, but I don't quite understand result X. I have already researched it and found…, but I can't reconcile it with my working diagnosis Y, because…. How would you explain result X?*

**P**roblem description	What problem do I encounter?
**O**ption overview	What options do I see for solving this problem?
**W**eighing options	How do I weigh these options in terms of pros and cons?
**E**xpressing preferred option	Given these pros and cons, what option(s) would I chose myself?
**R**equesting feedback information	'Could you give me feedback on this (i.e. elements P, O, W, and E)?'

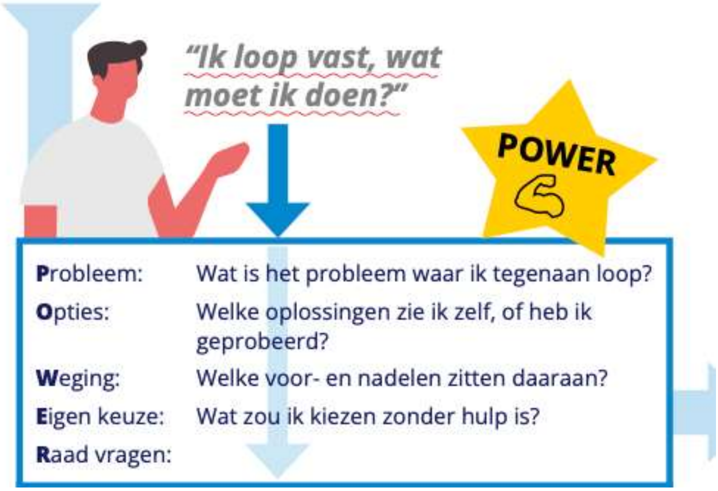

Used material in training in Dutch

(Image by Renske de Kleijn and Anne Geesink. Center for Academic Teaching and Learning, https://teaching-and-learning-collection.sites.uu.nl/knowledge_item/informatierijke-feedbackvragen/)

Session 3. Obtaining specific feedback, ask feedback on a learning goal using CLOSER(1)

The teachers discuss how asking CLOSER questions can assist students in receiving specific feedback as the CLOSER question guides students to ask feedback about a specific learning goal. An example of a CLOSER question is being discussed with the students and subsequently they practice formulating CLOSER questions during a simulation exercise. *Example: I would like to learn how to conduct ward rounds in a more structured manner. For the past week, I have been practicing with the structure used by the resident, which has been helpful, but I still find it quite challenging. I would like to know to what extent I am now conducting ward rounds in a structured way, so that I don't forget anything important and work efficiently. Could you give me feedback on this after the ward round?*

**C**urrent performance	My current performance, does (not) reflect the best I could do without additional assistance, because…
**L**earning **O**bjective	I would like my work to be evaluated on the following learning objective(s)…
**S**elf-**E**valuation	This is what I think I did well and what I would need to further improve
**R**equesting feedback information	'Could you give me feedback on this (i.e. elements C, LO, and SE)?'

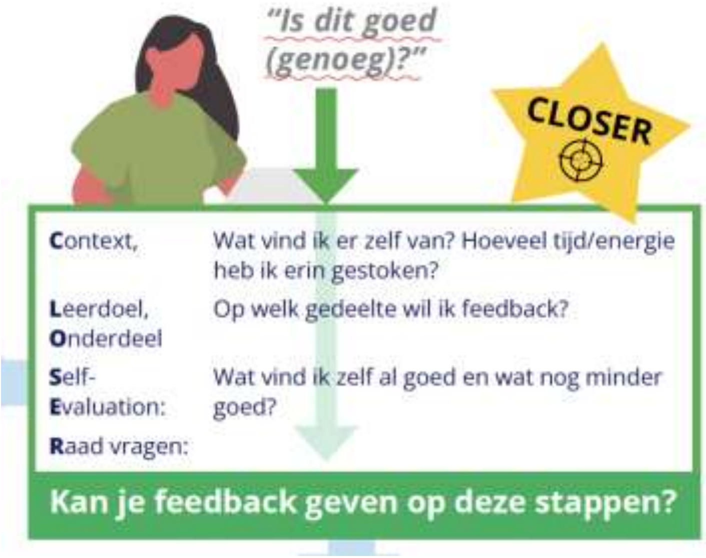

Used material in training in Dutch

(Image by Renske de Kleijn and Anne Geesink. Center for Academic Teaching and Learning, https://teaching-and-learning-collection.sites.uu.nl/knowledge_item/informatierijke-feedbackvragen/)

Brief explanation of the Westerveld Framework used in session 3 Feedback

The Westerveld Framework, based on research from Tielemans et al (2023)[1], was shared with students during session 3 to provide them with tips and trics on how to actively engage in feedback dialogues during clerkships (click here for the Westerveld Framework). The Westerveld Framework was developed to foster feedback dialogues in interprofessional healthcare settings. It describes seven criteria central to effective feedback dialogues: open and respectful, relevant, timely, dialogical, responsive, sense-making, and actionable. For each criterion, tips and trics are described for both the feedback user (those who receive feedback information, often the student during clerkships), and feedback giver (those who provide feedback information, often the workplace supervisors). This framework shows how both the feedback user and the feedback giver share a responsibility for having effective feedback dialogues, that these roles can switch, and it explains per role what this responsibility entails. Additionally, the framework incorporates specific interprofessional elements that address and provide solutions for overcoming common barriers to feedback dialogues within an interprofessional healthcare context. Students can use the framework to learn (a) how to seek feedback from various interprofessional partners, and (b) what they can do to effectively make sense of and use feedback. During the training, this tool was used to encourage students to become feedback users and show how they could take agency in their feedback process and what they might expect from their feedback givers. While the emphasis in the training was on encouraging students to become feedback users, the Westerveld Framework can also help students to prepare for their role as feedback givers.

[1]Tielemans et al (2023): Tielemans C, de Kleijn R, van der Schaaf M, van den Broek S, Westerveld T. The Westerveld framework for interprofessional feedback dialogues in health professions education. Assess Eval High Educ. 2023;48(2):241–257. DOI: https://doi.org/10.1080/02602938.2021.1967285

.

Session 4. Reflecting on your learning progress, at which station are you?

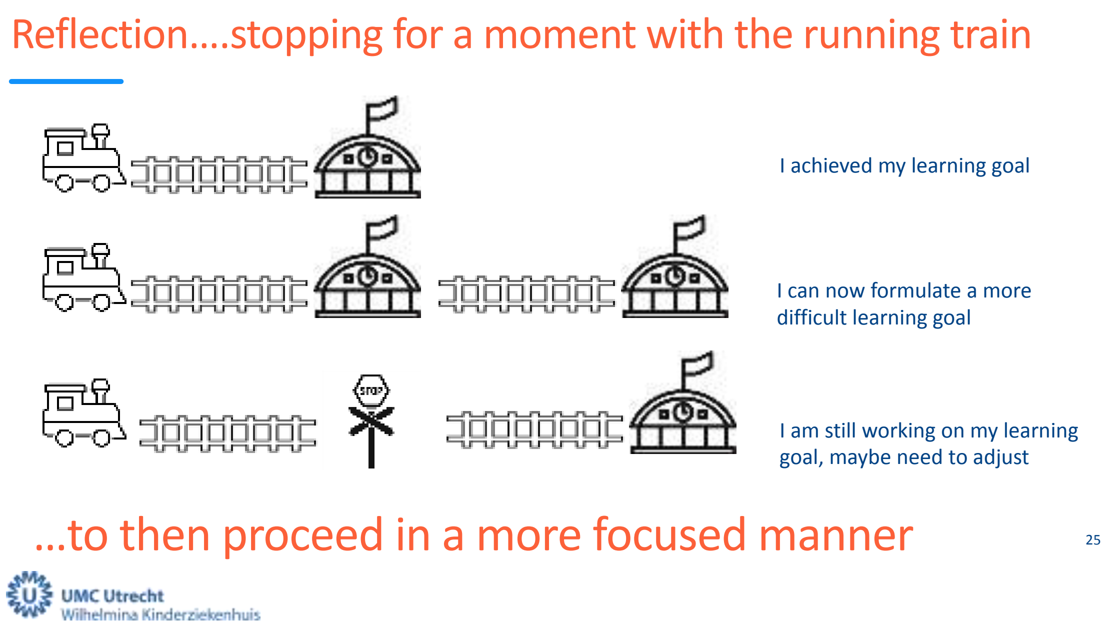

Students were asked to reflect on these statements and then walk to one of the abovementioned stations in the room and discuss their progress and what influenced this.

Statements:
1. I can formulate a learning objective that is appropriate to the specialty I do my clerkship in and that I can meet within the time frame I have

2. I can briefly and effectively introduce myself in the workplace so that everyone knows why I am there

3. I can ask through on feedback when I don't immediately see how to apply the feedback (take action)

Example of a fidelity form

This fidelity form was translated into English to serve as Supplementary data for this article.

**Fidelity-items[1,2]**	**Go through the following questions for each training activity**	**Introduction**	**Theory: Informal workplace learning**	**Group work: Informal learning activities**	**Theory: Learning Goals**	**Plenary simulation**	**Summary**	**Transfer Task**	**Explanation of workplace assignments**
**Adherence**									
Content	Have the intended learning objectives of this activity been addressed?								
Coverage	Which components of this activity are **not** given or adapted? Why?								
Duration	Does the actual time of this activity match with the intended time (longer/shorter)?								
Quality of delivery	Did you succeed in teaching the activity as it was intended?								
Intervention complexity & Facilitation strategies	Were the descriptions in the teacher guide and on the slides specific enough to teach this activity as intended?								
Participant responsiveness	How was this activity received by students?								
**Finally, when you look at this training session as a whole:**									
Essential elements	What were the most essential elements of this training? What did you think students benefited the most from?								

The students’ evaluation form

This evaluation form was translated into English to serve as Supplementary data for this article.

Which group are you in?
O Group 1
O Group 2

Which sessions did you participate in?
O Session 1: Introduction to informal workplace learning and learning goals
O Session 2: Asking questions
O Session 3: Feedback
O Session 4: Reflecting

Circle: At the time of the training I was involved in my *second / third* clerkship

The following statements are about what the training has/has not helped you with.
Please indicate the extent to which you agree with the following statements:

Because of this training I am more confident…

Very disagree **Very agree**

…to take advantage of learning opportunities at clerkship
O O O O O
…to formulate and discuss learning goals at clerkship
O O O O O
…to ask questions at clerkship
O O O O O
…to ask feedback at clerkship
O O O O O
…to adjust my learning at clerkships
O O O O O

The following statements are about the components of the training.
Please indicate the extent to which you agree with the following statements:

Very disagree **Very agree**

The training matched my expectations
O O O O O
The sessions matched with my challenges at clerkships
O O O O O
I can use the content of the sessions during my clerkships
O O O O O
The training provided new insights into learning during clerkships
O O O O O
The exchange with other students was useful
O O O O O
The workplace assignments were useful
O O O O O
The transfer tasks and debriefing moments were useful
O O O O O
There was an open and safe learning environment during the sessions
O O O O O
This training should be a mandatory part of the medical curriculum

O	O	O	O	O

The following statements are about the distribution of time during the training. Please indicate how you felt the distribution of time in the training was (too little, good, or too much):

	Too little	Good	Too much
In the sessions I was able to practice sufficiently with complex practical situations	O	O	O
In the sessions there was sufficient attention to the complexity of learning at clerkship	O	O	O
There was sufficient depth in the sessions	O	O	O

Circle: The training came *too early / on time / too late* in the medical curriculum

What did you appreciate about this training?

What would you like to change about this training?

Do you want to clarify any answers?

What do you *really* want to leave us with?

Thank you for your input and active participation in this training!
